# Cortical P1 Latency, Sound Field Audiometry, and Speech Audiometry in Children with Sequential Bilateral Cochlear Implants: A Comparative Pilot Study

**DOI:** 10.3390/jcm15207833

**Published:** 2026-10-10

**Authors:** Anna Waśniewska-Włodarczyk, Oskar Rosiak, Renata Pepaś, Kalina Niewiadomska, Wiesław Konopka

**Affiliations:** 1 Department of Otolaryngology, Polish Mother’s Memorial Hospital Research Institute, 93-338 Lodz, Poland; 2 Department of Pediatric Otolaryngology, Audiology and Phoniatrics, Medical University of Lodz, 91-738 Lodz, Poland

**Keywords:** cochlear implant, cortical auditory evoked potentials, sound field audiometry, speech audiometry

## Abstract

**Background/Objectives:** Objective assessment of auditory rehabilitation in children with cochlear implants (CIs) is challenging, as behavioral tests require cooperation that young children cannot always provide. The P1 component of the cortical auditory evoked potential (CAEP) is a marker of central auditory maturation, but few studies have compared it directly with behavioral measures in children with sequential bilateral CIs. **Methods:** Eight children (median age 8.4 years) with prelingual profound sensorineural hearing loss treated with sequential bilateral CI underwent CAEP (speech tokens/m/,/g/,/t/at 55 and 65 dB sound pressure level (SPL)), sound field audiometry and speech audiometry. An a priori physiological plausibility criterion excluded all detected responses in one child and one further response in a second child; correlations were therefore based on seven children and were assessed by Spearman rank correlation with exact permutation *p*-values. **Results:** P1 latency showed a frequency-specific association with sound field thresholds, strongest for the high-frequency/t/stimulus at 4 kHz (rho = 0.836, exact *p* = 0.056), which remained between +0.70 and +0.92 in leave-one-out analysis. Longer P1 latency tended to accompany poorer speech reception (mean latency at 65 dB SPL, rho = 0.679, exact *p* = 0.110), but this association was unstable, and no consistent relationship with duration of implant use was found Two coefficients reached nominal significance at 55 dB SPL but did not persist at 65 dB SPL, no other association was significant. **Conclusions:** In this pilot study P1 latency was associated with to frequency-matched aided thresholds, whereas its relationship with speech reception was directional but unconfirmed. CAEP may complement behavioral testing in this population, but larger longitudinal studies using sensation-level-matched stimulation are required.

## 1. Introduction

Cochlear implantation (CI) is a well-known method of treatment for children with profound sensorineural hearing loss. It significantly improves the quality of life across multiple domains, including social life, educational achievement, and psychological development. Despite its documented benefits, objective assessment of post-implantation auditory rehabilitation is still a clinical challenge, particularly in the pediatric population. Standard clinical evaluation relies mostly on behavioral speech perception tests; however, these measures require active cooperation from the patient, which limits their usefulness in infants, young children, and individuals with additional disabilities [1,2,3]. A further challenge concerns the assessment of central auditory development. While the auditory brainstem response (ABR) reflects the integrity of the subcortical auditory pathway, cortical auditory evoked potentials (CAEPs) provide an objective measure of how higher cortical centers process speech sounds [4,5].

The main components of the CAEP waveform are the P1, N1, and P2 peaks, which reflect stages of cortical auditory processing. The P1 component is a peak, where decreases systematically with increasing age. It reflects progressive maturation of the central auditory pathways, speech perception in children, and behavioral hearing thresholds [1]. In young children, normative P1 latency ranges from 100 to 200 ms, decreasing to 40–60 ms in adults [1,5,6,7]. This age-dependent reduction in P1 latency is thought to reflect increased efficiency of synaptic transmission and myelination within the auditory cortex. In CI users, the processing delay introduced by the sound processor is added to the neural conduction time, so P1 latencies shorter than approximately 50 ms are physiologically implausible even in adolescents and should raise suspicion of stimulus-related artifact. Due to its consistent presence across age groups and its sensitivity to auditory experience, P1 latency has been established as a non-invasive biomarker of central auditory pathway maturation in children with hearing loss using CIs [4].

Conventional behavioral audiological assessments, including sound field audiometry and speech audiometry, require active patient cooperation, which limits their applicability in younger children. Moreover, these measures do not directly assess the maturation of higher auditory cortical structures and usefulness of CI. CAEPs offer a complementary objective approach to evaluate central auditory pathway development and might be especially useful in patients with CI [1,8].

Despite growing interest in the objective assessment of auditory rehabilitation in children with CIs, there is a lack of studies comparing CAEP results with sound field audiometry and speech audiometry in children with sequential bilateral CIs. Previous studies have typically evaluated only two types of examination or focused on children with unilateral CI. Furthermore, few studies have used frequency-matched analysis, comparing P1 latency for/m/,/g/, and/t/stimuli with hearing thresholds at corresponding frequencies in sound field audiometry [2,9].

The aim of this study was to compare the results of three audiological examinations, CAEP, sound field audiometry, and speech audiometry in children with sequential bilateral CI, and to evaluate the correlations between P1 latency, hearing thresholds, and speech perception scores in this patient group.

## 2. Materials and Methods

This was a retrospective analysis of audiological data collected during routine clinical care. The study included 8 children, 5 girls and 3 boys, hospitalized at the Department of Otolaryngology, Polish Mother’s Memorial Hospital Research Institute, Łódź, Poland, with prelingual profound sensorineural hearing loss who underwent sequential bilateral CI. The etiology of hearing loss varied among patients, as did the age at implantation. The median age of the patients was 8.4 years (range 5.6–12.5). The better-performing ear was the first-implanted side in six of the eight children. Characteristics of patients is in Table 1.

### 2.1. CAEP

CAEPs were recorded using the Interacoustics Eclipse system with the Aided Cortical module. The active electrode was placed at the vertex (Cz), the ground electrode on the high forehead, and reference electrodes over the mastoid prominence behind each ear. In this examination patients had both implants on, reflecting the child’s everyday listening condition.

Three speech tokens/m/,/g/, and/t/(ManU-IRU) were used as stimuli, carrying dominant spectral energy in the low- (240–611 Hz), mid- (1084–1717 Hz), and high-frequency (2828–4468 Hz) regions, respectively. Stimuli were presented in the free field through a loudspeaker positioned at 0° azimuth, 1.5 m from the child, who remained seated and watched a silent video to maintain a calm and alert state. Each stimulus was presented at two intensity levels, 55- and 65-dB SPL.

The presence of a cortical response was determined automatically using the Fmpi detection algorithm implemented in the Eclipse system. Fmpi estimates a signal-to-noise ratio by comparing the variance of the averaged waveform with the estimated residual noise, incorporating all measured data points of the waveform and taking each listener’s individual EEG activity into account. A response was considered present when the Fmpi confidence level reached 95%. Only responses meeting this criterion were included in the analysis, and the latency of the P1 component was recorded for each reliably detected response. Before the correlations were computed physiological plausibility criterion was applied to all Fmpi-detected responses. Responses with P1 latencies below 50 ms were excluded a priori, since such values fall below the adult norm even before the sound processor delay is added, and were considered potentially contaminated by stimulus-related artifact.

### 2.2. Sound Field Audiometry

Sound field audiometry was performed in a sound-treated room using frequency-modulated (warble) tones presented through two loudspeakers positioned at ±45° azimuth, at a distance of 1.5 m from the patient. Behavioral hearing thresholds were determined at 500, 1000, 2000, and 4000 Hz using a standard clinical bracketing procedure (10 dB down/5 dB up). Each CI was assessed separately by testing with one speech processor activated at a time while the contralateral processor was switched off. For each patient, the thresholds of the better-performing ear were used for subsequent analysis. Results were expressed as thresholds in dB HL for each frequency and as a four-frequency average.

### 2.3. Speech Audiometry

Speech audiometry was performed in a sound-treated room using the Polish monosyllabic word lists developed by Pruszewicz, presented through two loudspeakers positioned at ±45° azimuth, at a distance of 1.5 m from the patient. Age-appropriate word list versions were selected according to each child’s age. As with sound field audiometry, each CI was assessed separately by testing with one speech processor activated at a time while the contralateral processor was switched off, and the results of the better-performing ear were used for subsequent analysis. In all patients, the same ear was identified as better-performing in both sound field and speech audiometry, ensuring consistency across measures. Words were presented across an individually adjusted range of intensity levels, adapted to each child’s ability to cooperate. At each level, the percentage of correctly repeated words was calculated. The speech reception threshold (SRT) was defined as the intensity level at which the patient correctly repeated 50% of the presented words, determined by linear interpolation from the individual articulation (performance–intensity) curve.

### 2.4. Statistical Analysis

Given the small sample size, all analyses were exploratory and hypothesis-generating. Associations were assessed using Spearman rank correlation with mid-ranks for ties, with two-sided *p*-values obtained by exact permutation. Responses not meeting the criteria in Section 2.1 were treated as missing and correlations computed by pairwise deletion, with the number of pairs reported throughout; note that with four pairs the smallest attainable exact *p*-value is 0.083. Mean P1 latency was defined, for each patient and intensity level, as the arithmetic mean of the latencies available at that level. The frequency-matched analysis was specified in advance:/m/with 500 Hz,/g/with the mean of 1 and 2 kHz, and/t/with 4 kHz. Agreement between methods was examined descriptively by ranking patients on mean P1 latency at 65 dB SPL, four-frequency average sound field threshold, and SRT, all sharing the same direction (lower = better), and each primary correlation was recomputed omitting one patient at a time. No correction for multiple comparisons was applied; significance was set at *p* < 0.05, with *p*-values of 0.05–0.10 reported as trends. Analyses used GraphPad Prism 10.

## 3. Results

The results obtained from all patients in sound field audiometry, speech audiometry, and CAEP are presented in Table 2. All six responses recorded in patient 7 (36–46 ms) and one response in patient 6 (65 dB SPL/g/, 30 ms) fell below the plausibility criterion defined in Section 2.1 and were excluded. The correlation analyses were therefore based on seven children. Mean P1 latency was calculated from three stimuli in three patients, two in two patients and one in two patients at 65 dB SPL, at 55 dB SPL the corresponding figures were three, two and one, with no analyzable response in one child. Word recognition scores clustered at the upper end of the scale: five of the seven analyzed children reached 100% correct at the highest level tested and three were already at 100% at 65 dB HL, leaving too little variance for rank-based analysis. The SRT, calculated from the whole articulation curve, gave a distinct value for every child.

### 3.1. CI-2 Use Duration vs. P1 Latency

**Figure 1:**
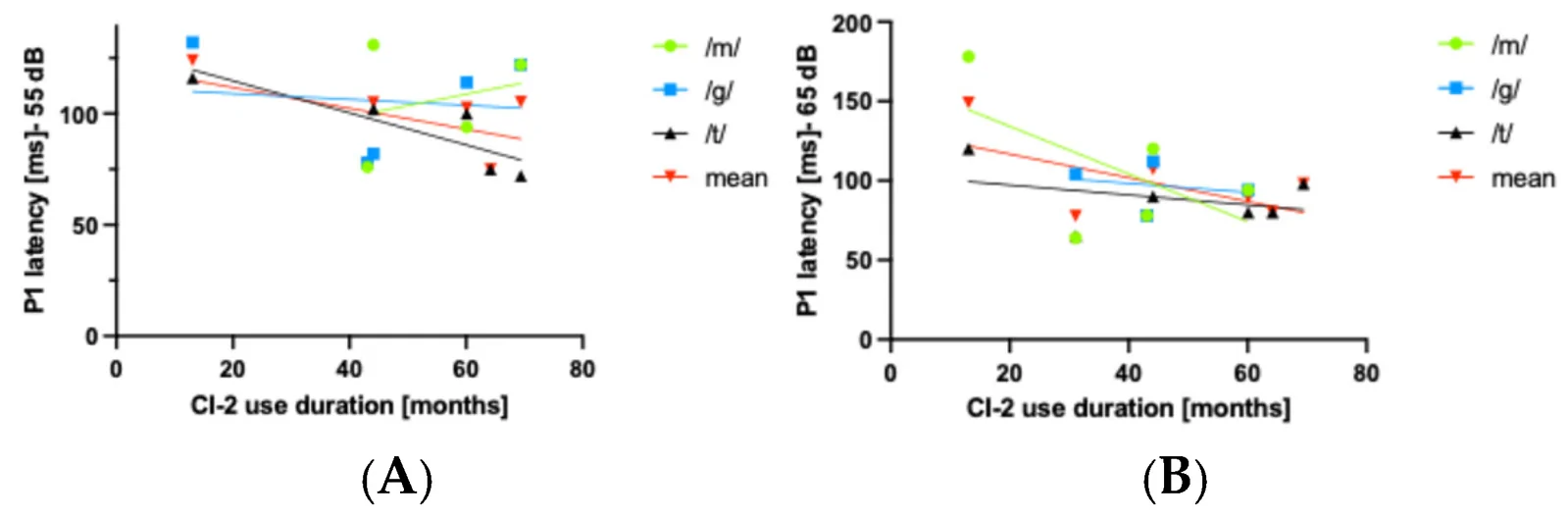
CI-2 use duration vs. P1 latency at 55 dB SPL (**A**) and 65 dB SPL (**B**). Lines show linear trends for groups with n ≥ 3.

P1 latency was correlated with the duration of second-implant (CI-2) use, separately for each stimulus (/m/,/g/,/t/) and for the mean of available responses, at both 55 and 65 dB SPL (Table 3 and Figure 1). Duration of second-implant use was chosen because cortical responses were recorded with both processors active, so that the second implantation determines how long the child had been listening bilaterally at the time of recording.

Apart from a single exception, the correlations between P1 latency and CI-2 use duration were all non-significant and close to zero, ranging from +0.400 to −0.257 with inconsistent direction across stimuli and intensity levels. The exception,/t/at 55 dB SPL (rho = −1.000, exact *p* = 0.017, n = 5), was in the direction reported previously, but the corresponding pair at 65 dB SPL, which included one additional patient, showed no association (rho = −0.058, n = 6). No relationship between P1 latency and cumulative implant use could therefore be demonstrated in this cohort, although the available sample size was too small to detect associations of the magnitude reported in larger studies. Mean P1 latency at 65 dB SPL was likewise unrelated to age at examination (rho = −0.071, *p* = 0.906), age at CI-1 implantation (rho = +0.286, *p* = 0.556), age at CI-2 implantation (rho = +0.036, *p* = 0.951) or inter-implant interval (rho = −0.464, *p* = 0.302). The youngest child (5.6 years) had one of the shortest latencies, so P1 latency did not track chronological age in this cohort. Because the better-performing ear was the first-implanted side in five of the seven analyzed children, we repeated the analysis using duration of first-implant use. All eight coefficients were negative, ranging from −0.348 to −1.000, and one reached significance (mean latency at 55 dB SPL, rho = −0.886, exact *p* = 0.033, n = 6). This is exploratory: duration of first-implant use is strongly related to age at examination (rho = 0.750), the coefficients are not mutually independent, and no correction for multiple comparisons was applied.

### 3.2. SRT vs. P1 Latency

**Figure 2:**
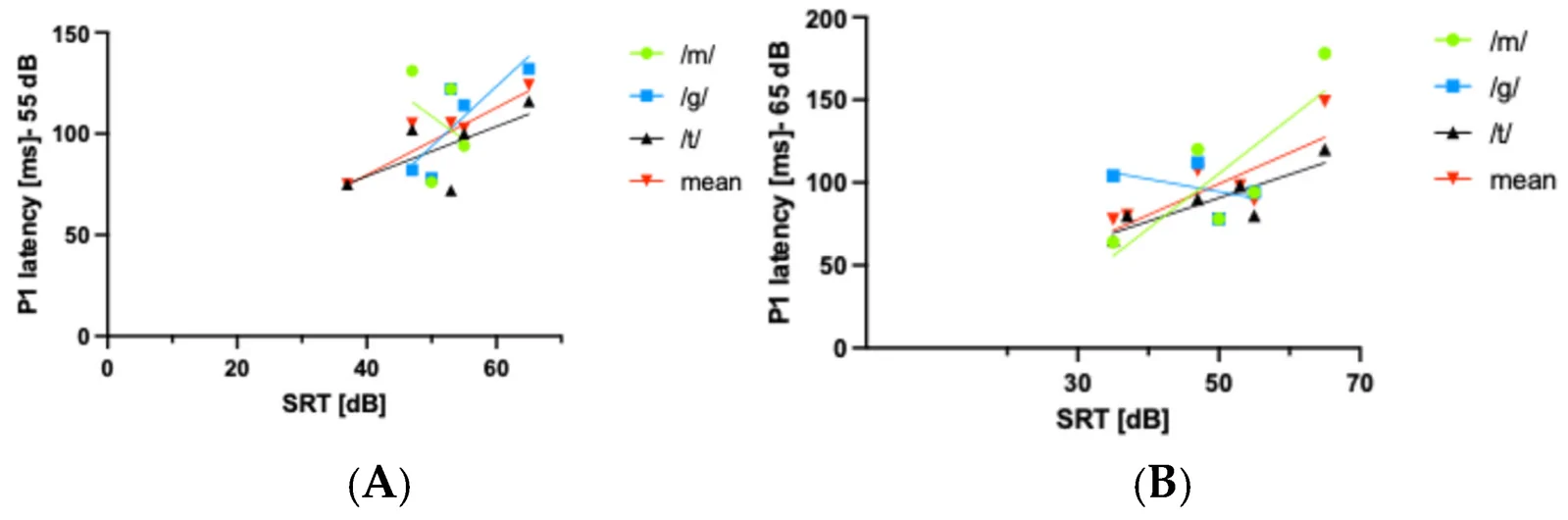
SRT vs. P1 latency at 55 dB SPL (**A**) and 65 dB SPL (**B**). Lines show linear trends for groups with n ≥ 3.

P1 latency was correlated with the SRT, separately for each stimulus and for the mean, at both intensity levels (Table 4). In contrast to the use-duration analysis, the correlations between P1 latency and SRT were predominantly positive (six of eight coefficients), indicating that longer P1 latencies tended to accompany higher (poorer) SRT. The association was most apparent for the/t/stimulus at 65 dB SPL (rho = +0.754, exact *p* = 0.111) and for the mean latency at the same level (rho = +0.679, exact *p* = 0.110). Although these values represent moderate-to-strong rank correlations, none reached statistical significance or the pre-specified trend threshold of *p* < 0.10, and the results are therefore best interpreted as a consistent directional tendency rather than a confirmed association (Figure 2). The two negative coefficients (/m/at 55 dB SPL, rho = −0.400;/g/at 65 dB SPL, rho = −0.600) were both derived from only four observations, the smallest subsets in the analysis; at this sample size the minimum attainable two-sided exact *p* is 0.083, so no coefficient based on four pairs can reach significance.

### 3.3. Frequency-Matched: P1 Latency vs. Sound Field Thresholds

Each stimulus was matched to the sound field threshold in its corresponding frequency range:/m/to 500 Hz,/g/to the mean of 1 and 2 kHz, and/t/to 4 kHz (Table 5).

**Figure 3:**
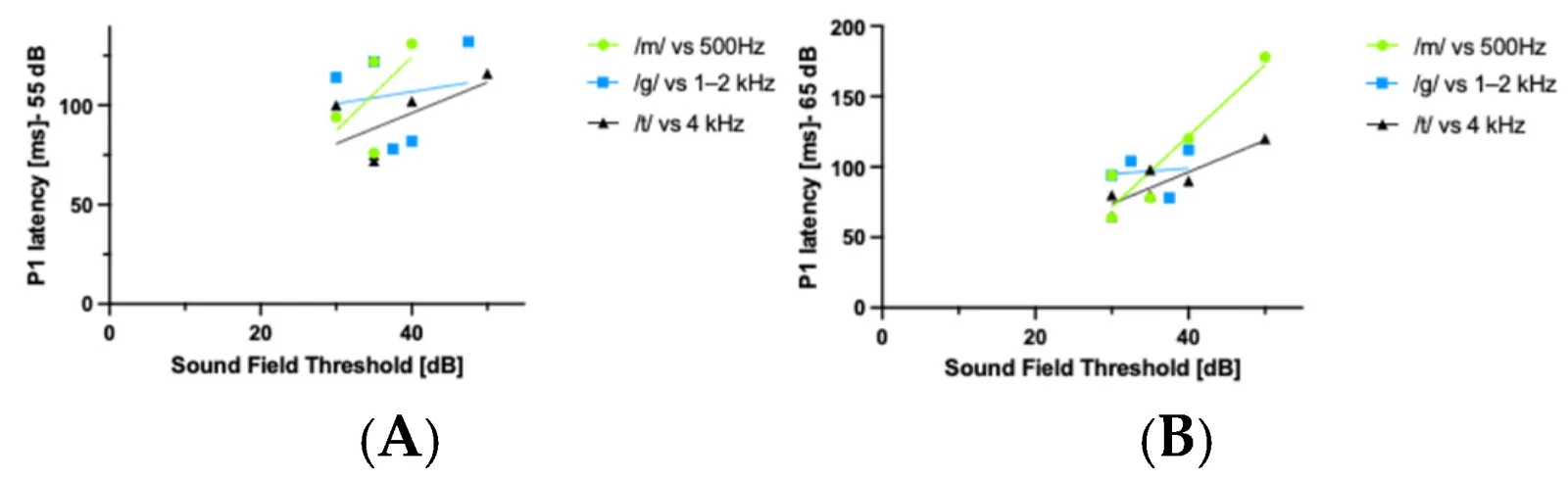
Frequency-matched P1 latency vs. sound field thresholds at 55 dB SPL (**A**) and 65 dB SPL (**B**). Dashed lines show linear trends for groups with n ≥ 3.

The frequency-matched analysis revealed a frequency-dependent pattern. The low-frequency/m/stimulus and the high-frequency/t/stimulus both showed positive correlations with their corresponding thresholds, and these were stronger at 65 than at 55 dB SPL, whereas the mid-frequency/g/stimulus showed only weak associations at either level (rho = +0.200 and +0.400). The strongest relationship of the entire analysis was the high-frequency/t/–4 kHz pair at 65 dB SPL (rho = +0.836, exact *p* = 0.056) (Figure 3). In leave-one-out analysis this coefficient remained between +0.703 and +0.921 regardless of which patient was omitted, indicating that it was not driven by a single observation, although it rests on only six pairs. It was the largest of the coefficients computed, no correction for multiple comparisons was applied, and replication in a larger sample is required. This correspondence is compatible both with a central account and with a purely audibility-based one, and the present design cannot distinguish between them.

### 3.4. Agreement Across the Three Assessment Methods

To examine whether the three assessment methods ordered patients consistently, patients were ranked on CAEP (mean P1 latency at 65 dB SPL), sound field audiometry (four-frequency average), and speech audiometry (SRT). All three measures share the same direction, with lower values indicating better performance, making the rankings directly comparable; ties were handled by mid-ranks (Table 6).

**Figure 4:**
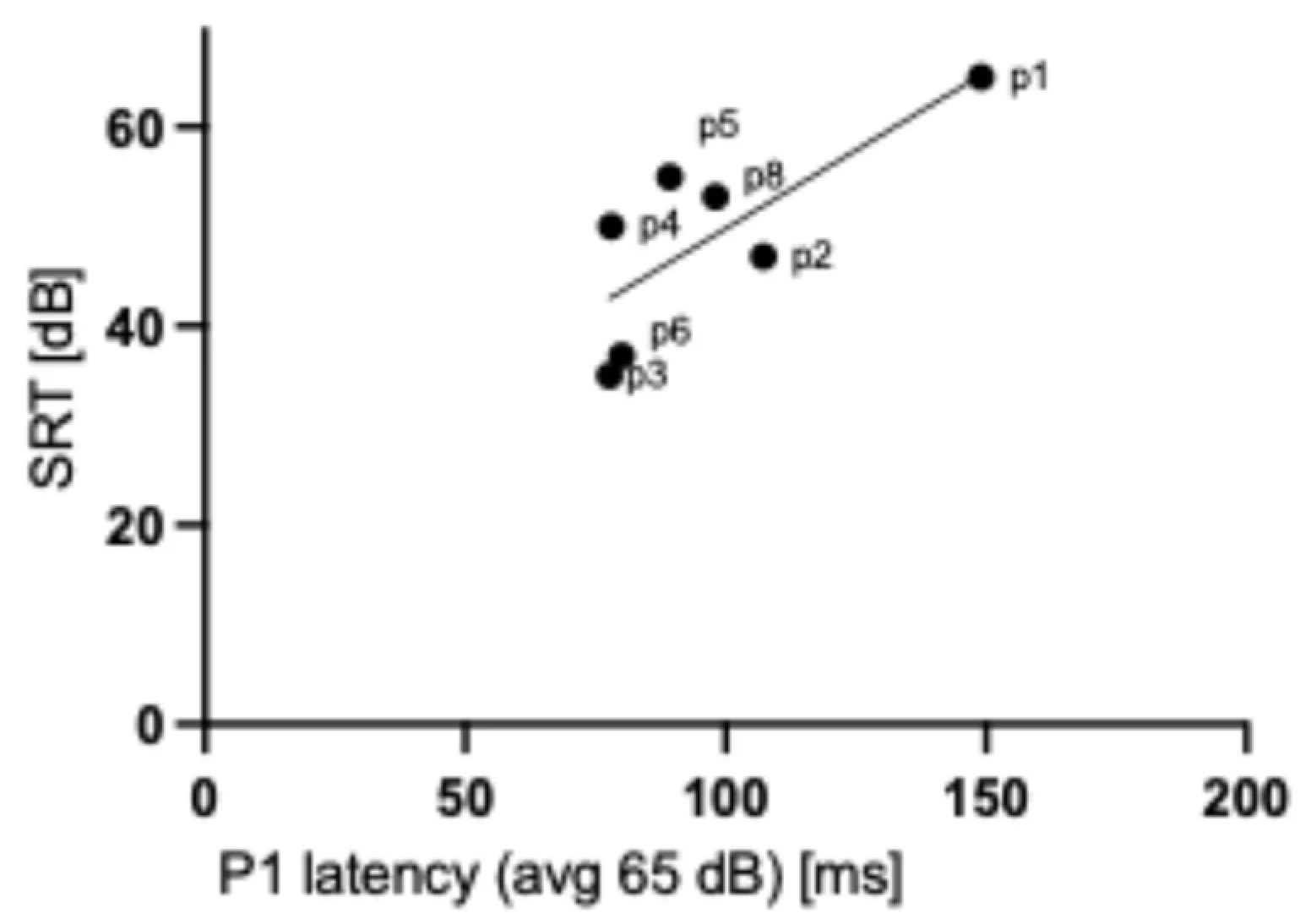
P1 latency (avg 65 dB SPL) versus speech reception threshold (SRT). rho = 0.679; exact *p* = 0.110.

**Figure 5:**
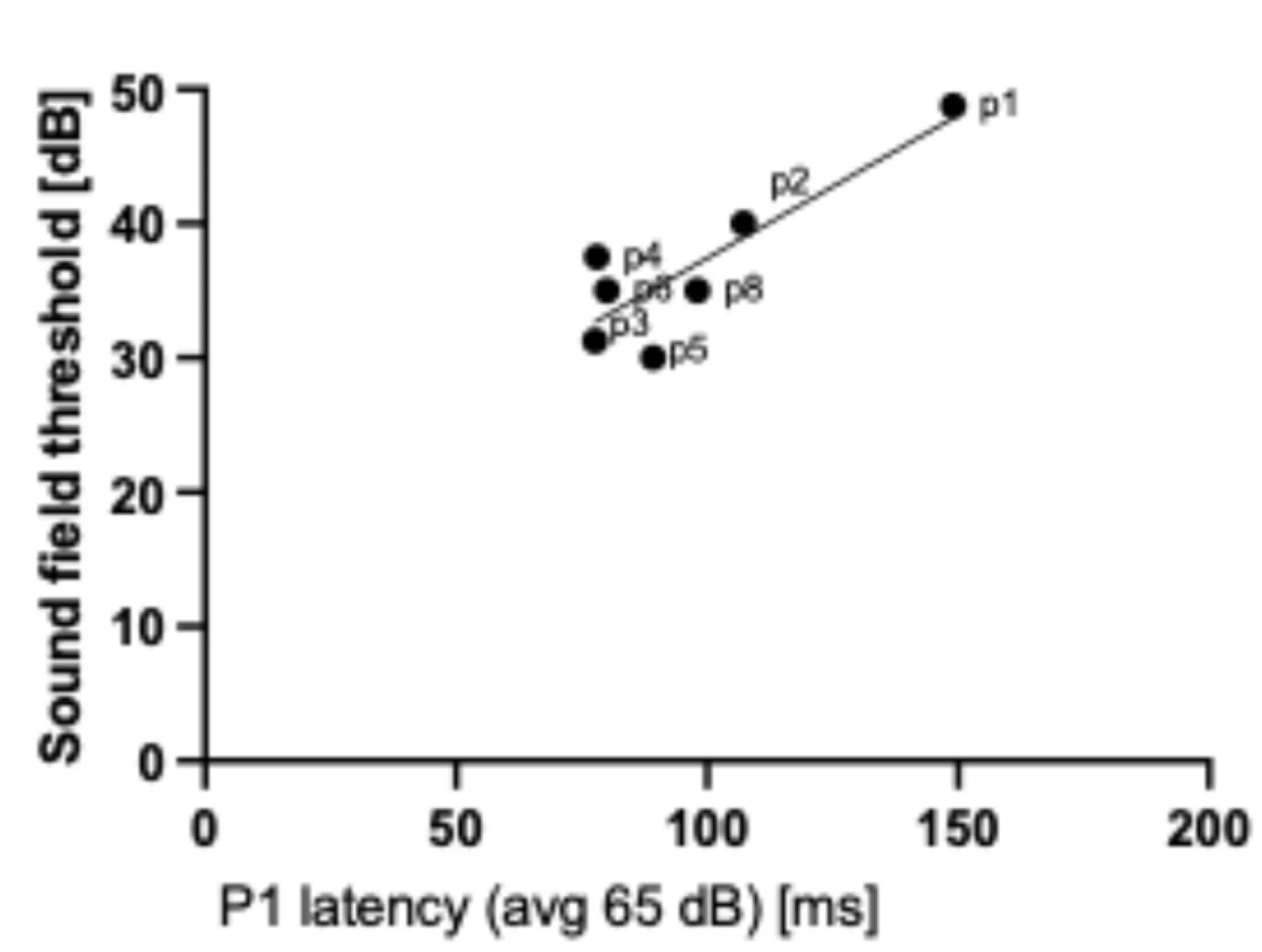
P1 latency (avg 65 dB SPL) versus sound field threshold (four-frequency average). rho = 0.613; exact *p* = 0.153.

**Figure 6:**
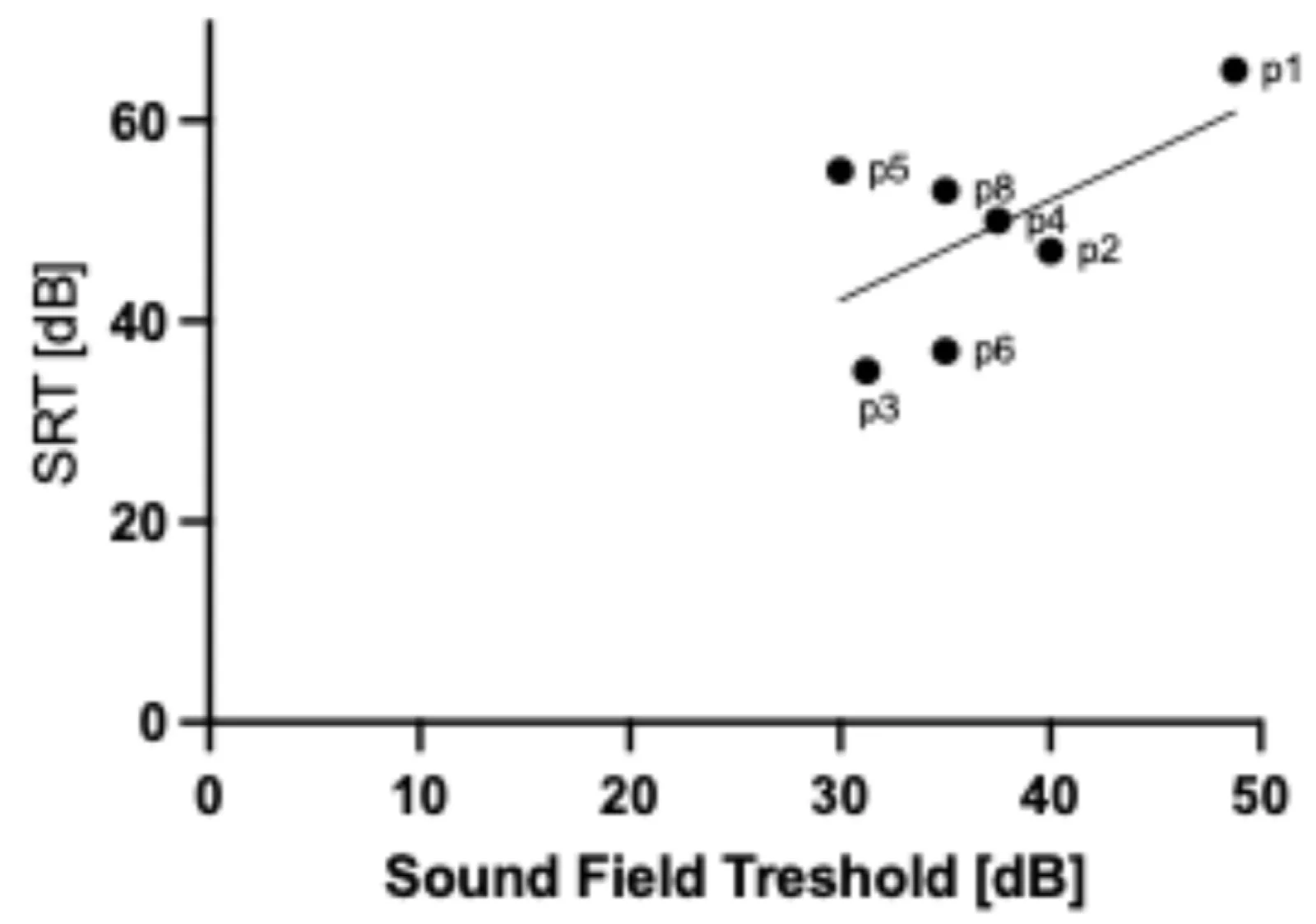
Sound field threshold (four-frequency average) versus SRT. rho = 0.270; exact *p* = 0.558.

Pairwise Spearman correlations (n = 7) showed that mean P1 latency at 65 dB SPL correlated with speech reception (CAEP vs. SRT: rho = 0.679, exact *p* = 0.110) and, more weakly, with sound field thresholds (CAEP vs. sound field: rho = 0.613, exact *p* = 0.153), whereas the two behavioral measures correlated only weakly with each other (sound field vs. SRT: rho = 0.270, exact *p* = 0.558). None of these coefficients reached significance, and in leave-one-out analyses all three proved unstable: CAEP versus SRT ranged from +0.486 to +0.886, CAEP versus sound field from +0.377 to +0.812, and sound field versus SRT from −0.174 to +0.696, depending on which patient was omitted. The ordering of the three coefficients therefore rests substantially on individual observations, most notably patient 1, who ranked last on all three methods, and does not support any conclusion as to whether P1 latency corresponds more closely to audibility or to speech reception. That child was extreme on several clinical variables simultaneously: the latest first implantation (5.6 years, compared with 0.8–2.9 years in the remaining children), the shortest duration of CI-2 use (13.1 months versus 31–69 months), the poorest aided thresholds (four-frequency average 48.75 dB HL versus 30–40 dB HL), the poorest SRT and the longest P1 latency, and was the only child with an acquired (ototoxic) etiology; the child was, however, neither the youngest nor the oldest at examination. The consistency of this profile across clinical and audiological measures explains why the agreement between methods depends so heavily on this single patient. These relationships are illustrated in Figure 4, Figure 5 and Figure 6.

A further sensitivity analysis excluded the patients whose mean P1 latency was based on a single stimulus. At 65 dB SPL the results were essentially unchanged: the correlation with SRT was +0.700 (n = 5) compared with +0.679 (n = 7), and with the four-frequency average sound field threshold +0.700 compared with +0.613; neither reached significance. At 55 dB SPL the correlation with SRT fell from +0.714 to +0.500. No imputation was attempted, since at this sample size any imputation model would rest on assumptions the data cannot support.

## 4. Discussion

The main purpose of our study was to investigate the relationship between behavioral hearing measures (sound field and speech audiometry) and CAEP in children with sequential bilateral CIs. Despite the established value of P1 latency as a marker of central auditory maturation, relatively few studies have directly examined its relationship with behavioral hearing outcomes in CI users [6,9].

Our finding that longer P1 latency was associated with poorer speech reception, although it did not reach even the pre-specified trend threshold, is directionally consistent with previous reports linking P1 latency to behavioral outcomes in children with CIs. Speech audiometry indexes the level at which speech material becomes intelligible and is therefore functionally closer to everyday communication than tone thresholds, although the SRT used here remains a threshold rather than a suprathreshold measure of recognition. Frånlund et al. [6], in a cohort of children with CIs, reported a significant negative correlation between P1 latency and receptive language scores on one language measure (Reynell III), though not on another (TROG-2). In experienced adult CI users, Kelly et al. found that better cortical potential measures, were associated with better speech perception [10]. Xiong et al., in a study of 48 children with CIs, found that P1 latency, in electrically evoked CAEP, was significantly negatively correlated with both auditory performance and speech intelligibility scores. It means that shorter P1 latency was associated with better auditory and speech outcomes [2]. Moreover, Visram et al. found that intracochlear eCAEP thresholds were close to behaviorally measured levels [11]. However, that approach relied on direct electrical stimulation of individual electrodes, whereas the present study assessed acoustically evoked cortical responses in the sound field, which more closely reflect everyday listening through the implant.

Across the three stimuli, mean P1 latency was longest for the low-frequency/m/stimulus (106.3 ms) and progressively shorter for the mid-frequency/g/(101.8 ms) and high-frequency/t/(90.7 ms) stimuli. This ordering is consistent with the pattern reported by Baydan et al. [9] in children with hearing aids, in whom the/m/stimulus also elicited the longest latency. Two mechanisms may plausibly contribute. First, the temporal envelopes of the stimuli differ:/t/is a burst with an abrupt onset whereas/m/rises slowly, and CAEP latency is known to depend on stimulus rise time. Second, in implant users the low-frequency channels of the sound processor involve longer analysis windows and therefore greater group delay. Cochlear travelling-wave mechanics, which are commonly invoked to explain frequency-dependent latency differences in acoustic hearing, cannot account for the present gradient, because the implant bypasses them. These values should be interpreted with caution: they pool the 55 and 65 dB SPL conditions from the same children, are based on partially non-overlapping subsets of patients for each stimulus, and the differences among stimuli were not formally tested. The ordering was also clearer at 65 dB SPL (106.8, 97.0 and 88.8 ms for/m/,/g/and/t/) than at 55 dB SPL, where/m/and/g/were essentially equivalent (105.8 and 105.6 ms).

Cortical responses were absent in a substantial proportion of conditions: 14 of 21 possible responses were detected at 55 dB SPL and 15 of 21 at 65 dB SPL. Detection rates were therefore comparable at the two levels, and in two patients fewer responses were detected at the higher level, so our data do not demonstrate a systematic level effect. CAEP detectability is known to depend on sensation level rather than on absolute stimulus level: Guo et al. [1], in children with hearing aids, showed that CAEPs are systematically undetectable below approximately 10 dB SL, and that an absent response most often reflects a small difference between the individual’s threshold and the stimulus level rather than a limitation of the method. Sensation level could not be equated in the present study, since stimuli were delivered at fixed levels in dB SPL while behavioral thresholds were expressed in dB HL, and this explanation therefore could not be tested here. The 65 dB SPL condition was adopted as the primary analytic level not because it yielded more detectable responses, but because it was the only level at which a mean P1 latency could be derived for all seven patients. Occasional absence of a response at suprathreshold levels is regarded as an expected phenomenon in pediatric electrophysiology, attributable to fluctuations in cortical arousal, subcortical gating and inter-individual variability, rather than to methodological shortcomings of the CAEP paradigm. Because all stimuli were presented at the same fixed level, children with better hearing heard them further above their threshold, so our main findings may reflect audibility rather than cortical maturation.

P1 latency was not significantly associated with the duration of second CI use. The single exception, a perfect inverse rank correlation for/t/at 55 dB SPL (rho = −1.000, exact *p* = 0.017, n = 5), was in the direction reported previously but did not persist at 65 dB SPL, where one additional patient contributed data. Most previous studies report that longer implant use is associated with shorter P1 latencies, although the strength of this association varies considerably, from strong (Jeong et al., r = −0.783) to weak (Xiong et al., r = −0.354) [2,12]. Our sample was too small to detect either: with seven patients the critical value of Spearman’s rho for a two-sided *p* < 0.05 is approximately 0.79, so even the strongest of these published associations would have failed to reach significance in the present cohort. In addition, P1 latency is dependent on age at implantation and the duration of auditory deprivation more than the cumulative implant use alone. In an exploratory analysis using duration of first-implant use, by contrast, all coefficients were negative and one reached significance, although that analysis is confounded with age at examination and no conclusion can be drawn from it.

In the present study, only the P1 component was analyzed. This choice reflects both the developmental characteristics of the cortical response and the specific population studied. In young children, the P1 component is the most prominent and reliably identifiable peak of the CAEP, whereas the later N1 and P2 components mature considerably later and are often absent or poorly formed before adolescence. Consistent with this, previous studies have reported very low detection rates for N1 and P2 in children, in contrast to the consistently present P1 [13,14].

## 5. Limitations

This study has several limitations. With seven analyzable patients, power was minimal (the critical value of Spearman’s rho at two-sided *p* < 0.05 is approximately 0.79), and leave-one-out analyses showed that the coefficients describing agreement between methods depended substantially on individual patients. A high proportion of Fmpi-detected responses did not enter the analysis, and the pattern of missing responses was not related to performance in a simple way: at 55 dB SPL no analyzable response was obtained in the child with the best SRT (patient 3) and only one in the child with the second best (patient 6), whereas responses were obtained in the poorest-performing child. Missingness therefore cannot be assumed to be at random in either direction, and mean P1 latency was derived from between one and three stimuli depending on the patient. Stimuli were presented at fixed levels in dB SPL rather than at equated sensation levels, so the correspondence between P1 latency and behavioral thresholds is compatible with a purely audibility-based account that the present design cannot exclude; cortical responses were also recorded with both processors active whereas behavioral testing was monaural. This mismatch reflects the retrospective nature of the data rather than a constraint of the method; in a planned prospective study both measures will be obtained in the bilaterally aided condition. Aided thresholds spanned a narrow, heavily tied range, and speech audiometry used individually adapted intensity ranges, limiting comparability between children. Finally, the study was cross-sectional and single-center, age was not statistically controlled despite being a strong determinant of P1 latency, the cohort was heterogeneous in age at implantation, inter-implant interval duration of CI-2 use and etiology, and 33 correlations were computed without correction for multiple comparisons. All analyses were therefore exploratory and should be addressed in larger, longitudinal studies using sensation-level-matched stimulation.

## 6. Conclusions

In children with sequential bilateral CI, CAEP could be recorded in the sound field and yielded P1 latencies that varied systematically with stimulus frequency. For the high-frequency/t/stimulus, P1 latency was associated with the aided threshold at the matched frequency; this was the most robust finding in the present cohort and remained stable in sensitivity analyses. By contrast, associations between P1 latency and speech reception or duration of implant use were weak and unstable and should not be regarded as established.

Because CI is increasingly performed in infants and toddlers, in whom behavioral audiometry is often not feasible, objective and cooperation-independent measures of auditory development are needed. The present findings are consistent with a complementary role for CAEP in this setting. They were, however, obtained in school-aged children who were able to complete behavioral testing, and extension to younger or less cooperative patients requires direct study. Larger, longitudinal investigations using sensation-level-matched stimulation are needed before CAEP can be recommended for routine clinical use in this population.

## Figures and Tables

**Table 1:** Characteristics of patients.

Patient	Age at Examination (Years)	Age at Implantation, CI1 (Years)	Side	Age at Implantation, CI2 (Years)	Side	Interval Between Implantation (Months)	CI2 Duration of Use (Months)	Better Hearing Ear	Etiology of Deafness
1	7.9	5.6	L	6.8	R	14.1	13.1	C1	Ototoxicity
2	6.6	0.9	R	2.9	L	24	44.1	C1	Genetic
3	5.6	1.5	L	3	R	18.2	31.1	C2	Maternal GDM
4	11	2.5	R	7.4	L	59.1	43.1	C1	Neonatal hyperbilirubinemia
5	8.6	1.2	R	3.6	L	29.1	60.1	C1	Congenital CMV
6	8.3	0.8	R	3	L	26	64.2	C2	Congenital, idiopathic
7	12.5	4.3	R	6.4	L	25	74	C1	Maternal GDM
8	9.6	2.9	R	3.8	L	10.1	69.4	C1	Congenital, idiopathic

R right, L left, CMV cytomegalovirus, GDM Gestational Diabetes Mellitus, CI-1 first-implanted side, CI-2 s-implanted side.

**Table 2:** Data from hearing examinations.

	Speech Audiometry [dB HL]								Sound Field Audiometry [dB HL]				CAEP [dB SPL]					
Patient	SRT (dB)	40 dB	50 dB	55 dB	60 dB	65 dB	70 dB	80 dB	0.5 kHz	1 kHz	2 kHz	4 kHz	55 dB/m/	55 dB/g/	55 dB/t/	65 dB/m/	65 dB/g/	65 dB/t/
1	65	0	20%	25%	30%	50%	60%	80%	50	45	50	50	-	132	116	178	-	120
2	47	20%	60%	75%	100%	100%			40	40	40	40	131	82	102	120	112	90
3	35	60%	80%	100%	100%	100%			30	30	35	30	-	-	-	64	104	65
4	50	0	50%	60%	70%	80%	90%	100%	35	35	40	40	76	78	-	78	78	-
5	55	0	30%	50%	70%	65%	60%	40%	30	30	30	30	94	114	100	94	94	80
6	37	70%	100%	100%	100%	100%			35	35	35	35	-	-	75	-	30	80
7	47	20%	60%	80%	100%	100%			35	25	25	25	40	36	44	42	46	42
8	53	-	40%	55%	70%	80%	90%	100%	35	35	35	35	122	122	72	-	-	98

SRT—speech reception threshold; t—high frequency, g—mid frequency, m—low frequency; CAEP—cortical auditory evoked potentials; HL—hearing level; SPL—sound pressure level.

**Table 3:** CI-2 use duration vs. P1 latency (n = 7).

Group	rho	*p* (Exact)	n
55 dB SPL/m/	+0.400	0.750	4
55 dB SPL/g/	0.000	1.000	5
55 dB SPL/t/	−1.000	0.017	5
55 dB SPL mean	−0.257	0.658	6
65 dB SPL/m/	−0.100	0.950	5
65 dB SPL/g/	0.000	1.000	4
65 dB SPL/t/	−0.058	0.933	6
65 dB SPL mean	0.000	1.000	7

t—high frequency, g—mid frequency, m—low frequency; SPL—sound pressure level.

**Table 4:** SRT vs. P1 latency (n = 7).

Group	rho	*p* (Exact)	n
55 dB SPL/m/	−0.400	0.750	4
55 dB SPL/g/	+0.800	0.133	5
55 dB SPL/t/	+0.500	0.450	5
55 dB SPL mean	+0.714	0.136	6
65 dB SPL/m/	+0.700	0.233	5
65 dB SPL/g/	−0.600	0.417	4
65 dB SPL/t/	+0.754	0.111	6
65 dB SPL mean	+0.679	0.110	7

t—high frequency, g—mid frequency, m—low frequency; SPL—sound pressure level.

**Table 5:** Frequency-matched P1 latency vs. sound field thresholds (n = 7).

Pair	rho	*p* (Exact)	n
55 dB SPL —/m/vs 500 Hz	+0.632	0.500	4
55 dB SPL —/g/vs mean(1.2 kHz)	+0.200	0.783	5
55 dB SPL —/t/vs 4 kHz	+0.667	0.267	5
65 dB SPL —/m/vs 500 Hz	+0.821	0.133	5
65 dB SPL —/g/vs mean(1.2 kHz)	+0.400	0.750	4
65 dB SPL —/t/vs 4 kHz	+0.836	0.056	6

t—high frequency, g—mid frequency, m—low frequency; SPL—sound pressure level.

**Table 6:** Patient rankings across the three methods (n = 7; 1 = best-performing).

Patient	CAEP Rank	Sound Field Rank	SRT Rank	Rank Sum
P1	7	7	7	21.0
P2	6	6	3	15.0
P3	1	2	1	4.0
P4	2	5	4	11.0
P5	4	1	6	11.0
P6	3	3.5	2	8.5
P8	5	3.5	5	13.5

## Data Availability

All data are placed in the manuscript.

## Abbreviations

The following abbreviations are used in this manuscript:

ABR	auditory brainstem response
CAEP	Cortical auditory evoked potentials
CI	Cochlear implant
SPL	Sound Pressure level
HL	Hearing level
SL	sensation level
SRT	Speech reception threshold
